# Effects of Slow Breathing Exercises on Heart Rate, Blood Pressure, and Quality of Life in Patients Undergoing Primary Percutaneous Coronary Intervention: Randomized Controlled Trial

**DOI:** 10.1007/s10484-026-09767-9

**Published:** 2026-02-02

**Authors:** Melike Çelik, Selda Çelik

**Affiliations:** 1 Nursing Department, Faculty of Health Sciences, Marmara University, Istanbul, Turkey; 2 Hamidiye Faculty of Nursing, University of Health Sciences, Istanbul, Turkey

**Keywords:** Blood pressure, Heart rate, Percutaneous coronary intervention, Quality of life, Slow breathing exercises

## Abstract

This study was conducted to determine the effects of slow breathing exercises performed following primary percutaneous coronary interventions (PCI) on the heart rate, blood pressure, and quality of life of patients. Data were collected from patients admitted to the cardiology inpatient clinic of a hospital in Türkiye after primary PCI between October 2023 and November 2024. Patients were randomly assigned to intervention (*n* = 34) and control (*n* = 33) groups. The intervention group performed slow breathing exercises twice daily for 10 min over an 8-week home follow-up period. Both groups monitored their heart rate and blood pressure at home during this time. Data were collected using a “Patient Information Form” and the “MacNew Heart Disease Health-Related Quality of Life Questionnaire” at discharge, as well as at the 4th and 8th weeks post-discharge. In all follow-up measurements, the quality of life of the patients in the intervention group was significantly better than that of those in the control group. Throughout the follow-up period, the antihypertensive medication doses of the patients in the control group needed to be increased significantly more. Slow breathing exercises increased the quality of life and reduced the heart rate and diastolic blood pressure values of patients following primary PCI. The exercises led to decreases in heart rate and blood pressure immediately after they were performed.

*Registry*: ClinicalTrials.gov, TRN: NCT06098222, Registration date: 5 October 2023.

## Introduction

Cardiovascular Diseases (CVD) are diseases of the heart and the circulatory system (WHO, 2025). An estimated 17.5 million people die of CVD-related causes worldwide, and 7.5 million of these deaths are caused by coronary artery diseases (CAD) and acute myocardial infarction (AMI). AMI is not only one of the diseases causing the higher rates of mortality in the world, but it also leads to significantly poorer health-related quality of life (Dias et al., 2014; Pikala & Maniecka-Bryła, 2017).

Acute coronary syndromes (ACS) are a set of conditions that result in the obstruction of blood flow in coronary arteries as a consequence of coronary plaque rupture, erosion, or thrombus formation, eventually leading to myocardial ischemia (Byrne et al., 2024). In addition to underlying atherosclerosis, ACS can also develop secondary to vasospasms. ACS cases are named based on the qualities of pain, Electrocardiography (ECG) results, and biochemical markers (Bergmark et al., 2022; Singh et al., 2023). An ST-Elevation Myocardial Infarction (STEMI), which is a form of ACS, is the most fatal class of atherosclerotic (CAD) with a poor prognosis and severe course (Byrne et al., 2024; Lawton et al., 2022). The most important symptom of a STEMI is ischemic chest pain lasting 10-to-20 min or longer, described by the patient typically as a feeling of burning, tightness, pressure, or weight and does not completely respond to nitroglycerin (Byrne et al., 2024; Singh et al., 2023; Van de Werf et al., 2008).

The purpose of the basic treatment of a STEMI is to achieve reperfusion as soon as possible. There are two methods of achieving reperfusion in the shortest time possible. These are primary percutaneous coronary interventions (PCI) and fibrinolytic/thrombolytic treatments. Due to better outcomes, primary PCI is preferred over fibrinolytic treatment. Primary PCI is defined as a Percutaneous Transluminal Coronary Angioplasty (PTCA) and/or Coronary Stent Implantation (CSI) procedure performed before or simultaneously with an interventional procedure (Byrne et al., 2024; TKD, 2022). Primary PCI is an effective intervention to facilitate and maintain the clearance of coronary arteries, and it allows practitioners to avoid some of the bleeding risks associated with fibrinolysis (TKD, 2022; Van de Werf et al., 2008). The guidelines of the European Society of Cardiology (ESC) recommend PCI within 90 min following the arrival of the patient for success in the treatment of STEMI cases (Byrne et al., 2024). The fact that this intervention is made in a vital organ and involves procedure-related uncertainties has been shown to cause anxiety and lead to changes in the vital signs of patients (Delewi et al., 2017; Francula-Zaninovic & Nola, 2018; Gallagher et al., 2010; Özer et al., 2009). In a cohort study conducted at 13 hospitals in China, only 30% of patients were found to have heart rate and blood pressure values in the target range after PCI (Wang et al., 2016). In another study, it was reported that after PCI, patients diagnosed with hypertension had a 10% higher rate of experiencing MI, and their risk of stroke was 2% higher (Lingman et al., 2011). In a four-year follow-up after PCI, the risk of CVD-related mortality increased two times in comparison to patients with heart rates below 70 bpm (Antoni et al., 2012). Changes in the vital signs of patients are a fundamental and modifiable risk factor for cardiac events in the later period following PCI (Zou et al., 2022). Therefore, after PCI, it is highly important to keep vital signs including the heart rate and blood pressure values of patients in balance.

According to the World Health Organization (WHO), quality of life refers to how a person perceives their life within the system of culture and values in which they live (Annaç, 2018). The physical, psychological, social, and occupational limitations occurring after ACS are effective in the disruption of the quality of life of the person (Demir & Özer, 2021; Özcanlı, 2012; Thompson & Roebuck, 2001). It was stated that MI influences the physiological, emotional, and social quality of life of individuals negatively for reasons such as its potentially progressive and severe complications and the likelihood of secondary ischemic events (Demir & Özer, 2021; Thompson & Roebuck, 2001). According to Failde and Soto, who examined the quality of life of patients three months after the discharge of patients from the hospital following ACS, concluded that the patients had permanently lower levels of quality of life after ACS (Failde & Soto, 2006).

Slow breathing exercises are a behavioral technique involving low respiratory rates. This technique allows the person to breathe effectively as they consciously inhale and exhale slowly (Ma et al., 2017; Yokogawa et al., 2018; Zou et al., 2022). Breathing exercises are easy to learn, and they can be practiced easily anywhere without needing any equipment (Duong et al., 2017). Slow and deep breathing exercises are effective in reducing sympathetic activity, increasing baroreflex sensitivity, and raising parasympathetic activity (Jerath et al., 2019; La Rovere et al., 2001; Zou et al., 2022). It was stated that keeping the respiratory rate at six breaths per minute for five minutes would support the formation of autonomic balance by increasing parasympathetic activation (Pramanik et al., 2009). In addition to their autonomic and central nervous system effects, slow breathing techniques (< 10 breaths/minute) have been associated with an increase in alpha waves and a decrease in theta waves in post-exercise Electroencephalography (EEG) studies. In a recent anatomical study, increased activities in cortical structures (e.g., prefrontal, motor, and parietal cortices) and subcortical structures (e.g., pons, thalamus, and hypothalamus) were measured in Magnetic Resonance Imaging. These results were associated with a state of psychological well-being (Zaccaro et al., 2018).

It is seen that breathing exercises have been utilized for several purposes such as decreasing the heart rate after PCI, controlling heart rate and blood pressure values in hypertensive patients, lowering atelectasis following surgical operations, improving pulmonary functions, alleviating symptoms and improving quality of life in asthma patients, strengthening respiratory muscles, gastroesophageal reflux cases, managing pain, and reducing anxiety, stress, and depression levels (Borge et al., 2014; Chandla et al., 2013; Eherer et al., 2012; Grams et al., 2012; Jones et al., 2003, 2015; Park et al., 2013; Santino et al., 2020; Schaffer & Yucha, 2004; Sorosky et al., 2008; Thomas et al., 2009; Valenza et al., 2014; Westerdahl et al., 2005; Zou et al., 2022). In a study conducted with CAD cases, slow breathing exercises were effective in reducing the diastolic blood pressure values of patients (D’silva et al., 2014). Patients who performed slow breathing exercises for eight weeks by resting five seconds after every six slow breaths showed lower systolic blood pressure and heart rate values (Jones et al., 2015). Zou et al. (2022) who instructed post-PCI patients to inhale through their nostrils for four seconds and exhale through their mouth slowly for six seconds, reported that the heart rates of the patients decreased (Zou et al., 2022). The monitoring and follow-up of the vital signs of patients after MI and PCI are important in terms of preventing the development of complications.

According to the National Center for Chronic Disease Prevention and Health Promotion in the USA, health-related quality of life has a clear influence on health (CDC, 2020). Health-related quality of life also involves the physical and psychological health perceptions of individuals and the individual-level correlations of these perceptions. Reduced health-related quality of life is an important outcome reported for CAD cases (Le et al., 2018). In the literature review, it was seen that to improve the vital signs and quality of life of patients undergoing PCI, various non-pharmacological methods such as training programs, massage, playing nature sounds, playing music, music therapy in different modes, and progressive relaxation exercises have been utilized (Carroll et al., 2017; Veremci, 2022; Yıldırım & Oğuz, 2022). It is believed that the results of this study will make innovative contributions to the relevant literature and guide future studies with their high level of evidence by determining whether slow breathing exercises performed following PCI procedures are effective in facilitating optimal heart rate and blood pressure values and increasing quality of life.

## Methods

This study was conducted with a randomized controlled design to determine the effects of slow breathing exercises performed by patients who underwent primary PCI with the diagnosis of STEMI after the procedure on their heart rates, blood pressure values, and quality of life. The hypotheses of the study were as follows:

H_1_: Slow breathing exercises performed after PCI reduce heart rate.

H_2_: Slow breathing exercises performed after PCI reduce systolic blood pressure.

H_3_: Slow breathing exercises performed after PCI reduce diastolic blood pressure.

H_4_: Slow breathing exercises performed after PCI increase quality of life.

### Design

This study is a prospective randomized controlled trial with an 8-week follow-up.

### Study Settings and Sampling

This study was carried out with patients who had undergone primary PCI at the cardiology inpatient clinics of a research and training hospital in Türkiye. The data were collected between October 2023 and November 2024. The population of the study consisted of patients who were admitted to the cardiology inpatient clinic of the aforementioned hospital within the dates of the study who had undergone primary PCI after STEMI, while the sample included those from the population who met the inclusion criteria and agreed to participate in the study. All patients received standard medical therapy following primary PCI.

A power analysis was carried out using the G*Power 3.1 program to calculate the sample size needed to conduct the study. The sample size calculation was based on the effect size determined using Cohen’s d method (Cohen, 1988). To estimate the effect size (d), we referred to the findings of D’silva et al. (2014), who reported a statistically significant difference of 7.5 mmHg in diastolic blood pressure between the control and intervention groups. Based on these findings, the effect size for our study was calculated as d = 0.878. At the planning stage of the study, a power analysis for a two-group comparison (t-test) was performed using the G*Power 3.1 software, assuming an effect size of 0.8, a 95% confidence interval (1-α), and 95% power (1-β). This analysis indicated that 35 participants per group, for a total of 70 participants, would be required. To account for potential dropouts, including a total of 80 participants was planned. Due to the fact that the data collection period had reached the end of the timeframe committed under the TÜBİTAK project, a post hoc power analysis was performed at the end of the study. With reference to the findings of D’silva et al. (2014), this analysis indicated that with a 95% confidence interval (1-α), α = 0.05, effect size d = 0.8, and 90% power (1-β), a total of 58 participants (29 per group) would be sufficient (D’silva et al., 2014). Patients who met the inclusion criteria were assigned to the intervention (*n* = 37) and control (*n* = 36) groups using the computer-assisted simple randomization method. The study was ultimately concluded with 34 participants in the intervention group and 33 in the control group (total *n* = 67). According to the post hoc power analysis, this sample size was sufficient, and there were no concerns regarding the adequacy of statistical power for the study.

### Randomization and Blinding

Randomization was performed independently of the researchers using a computer-generated block randomization method. The randomization sequence was created in blocks of 10 participants, ensuring equal allocation to the intervention and control groups. This approach was chosen to maintain numerical balance between the groups throughout the study period. The randomization list was used after participants were enrolled in the study, and allocation concealment was ensured. Group assignments were carried out using sequentially numbered, opaque, sealed envelopes. The researchers had no access to group allocation information prior to participant enrollment. All data were collected by a research nurse.

### Inclusion and Exclusion Criteria

The inclusion criteria were as follows:

- Being 18–65 years old,
- Having had a successful primary PCI procedure involving PTCA and CSI,
- Having a HR of > 60 bpm.
- Being a hypertension patient.
- Not having a mental or psychiatric illness,

The exclusion criteria were as follows:

- Having arrhythmias, physical limitations/respiratory distress severe enough to prevent breathing exercises,
- Having a disease that could raise HR (anemia, hyperthyroidism, hyperthermia, infection).

The drop-out criteria were as follows:

- Readmission to the hospital for any reason after discharge,
- Inability to complete the slow breathing exercises despite having received training,
- Not performing slow breathing exercises at home for one week during the follow-up period (intervention group),
- Not measuring heart rate and blood pressure at home for one week during the follow-up period.

Three patients from the intervention group and three patients from the control group were removed from the study. Patients were removed from the study if they were readmitted to the hospital for any reason after discharge, were unable to complete the slow breathing exercises despite training, requested to withdraw from the study, failed to perform slow breathing exercises at home for one week during the follow-up period (intervention group), or failed to measure their heart rate and blood pressure at home for one week during the follow-up period. Only patients who completed the study according to the criteria mentioned above were included in the analysis.

### Data Collection

A ‘‘Patient Information Form”, the “MacNew Heart Disease Health-Related Quality of Life Questionnaire (MacNew)”, a “Self-Monitoring Form”, and a “Patient Follow-Up Form” were used to collect data. During the slow breathing exercise training process, a “Slow Breathing Exercise Training Booklet” and a “Slow Breathing Exercise Video” were used.

### Patient Information Form

The form, which was created by the researcher, included questions about the sociodemographic characteristics (e.g., age, marital status, working status, education status), disease-related characteristics and risk factors (e.g., comorbidities, BMI, waist circumference, alcohol consumption and smoking status, medications used), and PCI-related characteristics (outcome of the procedure, blood vessel used during the intervention) of the patients. For the waist circumference measurements in the data collection form, after being informed about the procedure, the patient was asked to stand up. The clothes of the patient on their upper body were cleared by respecting their privacy. A measuring tape was wrapped around the waist of the patient at the level between the lowest rib and the crista iliaca (4 cm above the umbilicus). Finally, the clothes of the patient were fixed, and the patient was asked to lie down in a comfortable position (Enç & Uysal, 2019; Rudolf et al., 2007).

According to WHO, adults with BMI values of 25 kg/m^2^ or higher are considered overweight, while those with BMI values of 30 kg/m^2^ or higher are considered obese. Additionally, abdominal obesity is defined as a waist circumference of 88 cm or higher in women and 102 cm or higher in men. BMI is prevalently used to identify obesity based on the weight classification of WHO. BMI is calculated by dividing the weight (kg) of the individual by the square of their height (m) (BMI = kg/m^2^) (WHO, 2008, 2024).

### MacNew Heart Disease Health-Related Quality of Life Questionnaire

The MacNew Heart Disease Health-related Quality of Life Questionnaire was developed in 1991 by Oldridge et al. It was designed to develop how the physical, emotional, and social functions, and activities of daily living of MI patients are affected by CAD within a period of two weeks and evaluate post-MI quality of life. The validity and reliability study of the scale in Turkish was carried out by Daskapan et al. (Daskapan et al., 2008).

The results of the scale are interpreted based on its total score and the scores of its three dimensions. For this purpose, the average of the scores of all items in each dimension or the overall scale is calculated. This average varies between 1 and 7. Higher scores correspond to higher levels of quality of life. The scale consists of 27 items and three dimensions, which are the physical (*n* = 13), emotional (*n* = 14), and social (*n* = 13) domains, while some items are listed under multiple domains (Daskapan et al., 2008; Demir & Özer, 2021; Höfer et al., 2004). Cronbach’s alpha coefficient for the scale was reported as 0.93. In this study, Cronbach’s alpha coefficients for the scale were found to be 0.86 in the measurements made at discharge, 0.88 at the fourth week, and 0.91 at the eighth week.

### Self-Monitoring Form

The patients filled out this form daily to log their exercise activities after they performed slow breathing exercises for 10 min while watching the Slow Breathing Exercise Video twice a day. The patients also logged the results of the blood pressure and heart rate measurements they made following at least five minutes of rest and/or breathing exercises in the morning hours after a very light breakfast or two-four hours of fasting, at least three days a week. They were asked to measure their heart rate and blood pressure at home on a weekday independently of the slow breathing exercises and measure them consecutively before and after the exercises on the weekend. This form was checked by the physician who examined the patients when they arrived for their follow-ups, and it was collected at the end of the study.

### Patient Follow-Up Form

This form was used to record the heart rate and blood pressure values of the patients measured at the outpatient clinic when they arrived for their follow-ups at the end of the fourth and eighth weeks, as well as the changes -if any- in the drugs prescribed to them.

### Slow Breathing Exercise Training Booklet

This booklet was prepared in line with the relevant literature and used as educational material to train the patients about slow breathing exercises (Akademi, 2024; Zou et al., 2017, 2022). At the end of the training, the booklet was given to the patients so that they could use it at home while performing the slow breathing exercises. To make sure that the booklet did not have problems in terms of content, expressions, comprehensibility, or scientific rigor, it was submitted to the opinions of 10 experts, including two academician nurses, two clinic nurses, two physiotherapists, two physicians specializing in pulmonology, and two physicians specializing in cardiology, who assessed it independently. After receiving expert opinions, the protocol and booklet were rearranged and finalized.

### Slow Breathing Exercise Video

A video script was created by the researcher in line with the literature, and the researcher shot a short training video for the patients to watch while performing the slow breathing exercises (Akademi, 2024; Zou et al., 2017, 2022). To make sure that the video did not have problems in terms of content, expressions, comprehensibility, or scientific rigor, it was submitted to the opinions of 10 experts, including two academician nurses, two clinic nurses, two physiotherapists, two physicians specializing in pulmonology, and two physicians specializing in cardiology, who assessed it independently. The slow breathing exercise training was provided in a practical manner by showing the patients the video. The video was sent to the patients.

### Study Interventions

After patients who agreed to participate in the study were provided with information about the objective and methodology of the study, data were collected from those who gave their verbal and written consent. After more than 24 h following their PCI and/or on the next day, the “Patient Information Form” and the “MacNew Questionnaire” were filled out in the meeting room of the unit. The patients in the intervention group were given slow breathing exercise training. The training was provided using the “Slow Breathing Exercise Training Booklet” and the “Slow Breathing Exercise Video”. Before collecting data, the researcher, who is a nurse, received training as a ‘Breathing Therapy Practitioner’ in a program with an international accreditation certificate and the approval of the university. The training was given to the patients in a single one-hour session in the meeting room of the unit after more than 24 h following their PCI and/or on the next day. During the training session, the patients were instructed to rest quietly for 20 min and then achieve a respiratory rate of 6–8 breaths/minute by inhaling slowly through their nostrils for four seconds and exhaling slowly through their mouth for six seconds (Jones et al., 2015; Zou et al., 2022). Additionally, patients were allowed a short rest of approximately five seconds after every six breathing cycles to ensure comfort during the 10-minute exercise session. In the session, to determine whether the patient achieved the targeted respiratory rate, the chest and abdominal movements of the patient were observed, and a stopwatch was used. At the end of the training session, the patients were given the “Slow Breathing Exercise Training Booklet” printed in color.

After their discharge, the patients in the intervention group were asked to perform slow breathing exercises for 10 min twice every day for eight weeks. The “Slow Breathing Exercise Video” file was sent to the mobile phones of the patients. The patients were given the “Self-Monitoring Form” that they needed to fill out at home themselves. They were instructed to make heart rate and blood pressure measurements at home following at least five minutes of rest and/or breathing exercises in the morning hours after a very light breakfast or two-four hours of fasting, at least three days a week. They were asked to measure their heart rate and blood pressure at home on a weekday independently of the slow breathing exercises and measure them consecutively before and after the exercises on the weekend. The patients in the control group were also asked to make heart rate and blood pressure measurements at home following at least five minutes of rest in the morning hours after a very light breakfast or two-four hours of fasting, at least three days a week. Before their discharge, the patients were taught how to use the blood pressure measurement device that would be used to measure and record their blood pressure and heart rate values by demonstration, and the device was handed to them.

The patients were invited to attend physician follow-ups and re-evaluated using the data collection forms at the end of the fourth and eighth weeks. The heart rate and blood pressure values of the patients in the intervention group were measured at the office consecutively, before and after they performed the given exercises, and recorded. The slow breathing exercise training was also given to the patients in the control group after the study ended. All interventions, outcome assessments, and in-hospital vital sign measurements were performed by a single research nurse, who also delivered the slow breathing intervention and administered the MacNew Quality of Life Questionnaire.

The slow breathing exercises were performed daily, with the researcher providing instructions and monitoring adherence via patient logs, SMS reminders, and video calls. In-hospital vital signs were measured at each scheduled visit, and the MacNew Questionnaire was administered by the same researcher at predefined intervals throughout the study. To avoid device-related deviations in measurement values, the brand of blood pressure measurement devices that have been used in scientific studies before and are also recommended by the European Society of Hypertension were used in this study (Zhang et al., 2015). All patients were given the same brand and model of blood pressure monitor (Rossmax, Model CH155f), that made measurements on the upper arm procured using the project budget. Both the intervention and control groups received the same frequency and duration of contact throughout the study to ensure comparable attention and supervision.

### Data Analysis

The data collected in the study were analyzed using SPSS 27 (Statistical Package for the Social Sciences). As descriptive statistics, mean, standard deviation, median, minimum, and maximum values are presented for the quantitative variables, while frequency and percentage values are presented for the qualitative variables. The normality of the distributions of the data was checked using the Shapiro-Wilk test and Box Plots.

For the normally distributed data, Student’s t-tests were used to compare two independent groups, paired-samples t-tests were used to compare two measurements within the same groups, and repeated-measures analysis of variance (ANOVA) was used to compare three or more repeated measurements. For the non-normally distributed data, two independent groups were compared using the Mann-Whitney U test, and intragroup comparisons were made using the Friedman test. The relationships between the variables were analyzed using Pearson’s or Spearman’s correlation coefficients depending on the normality of their distributions. A Generalized Linear Model (GLM) was applied to evaluate the group and time interactions of scale scores and blood pressure outcomes. Chi-squared, Fisher’s exact, and Fisher-Freeman-Halton tests were used to compare qualitative data. The results of the analyses were evaluated within a 95% confidence interval and at a significance level of *p* < 0.05.

### Ethical Considerations

Ethical approval was obtained from the Clinical Studies Ethics Committee with the decision dated 31/01/2023 and numbered 2023/02/661, and institutional permission was obtained from the Istanbul Provincial Directorate of Health with the decision dated 21/03/2023 and numbered 2023/04. The principles of the Declaration of Helsinki were followed, and volunteers were provided with verbal and written information. The written consent of the participants was documented using the “Informed Consent Form”.

## Results

**Fig. 1:**
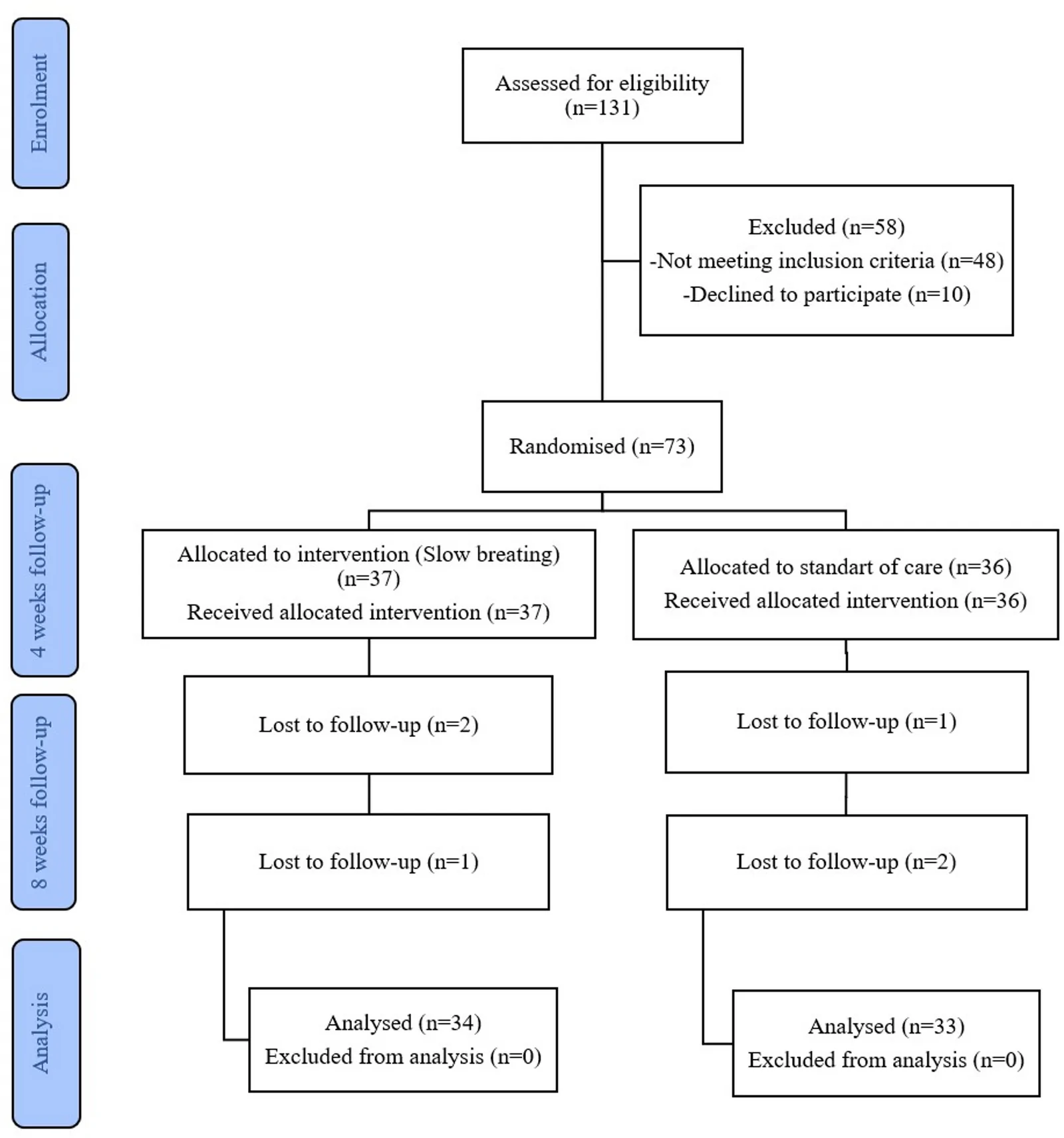
CONSORT 2025 Flow Diagram

Of the 131 participants recruited, 58 were excluded during the eligibility screening for various reasons. In total, 73 patients (intervention = 37, control = 36) who met the inclusion criteria of the study were reached. During the follow-up period, 3 patients in the intervention group and 3 in the control group needed to be removed from the sample according to the removal criteria. The study was completed with 67 patients in total, including 34 in the intervention group and 33 in the control group. The flow diagram of the study is presented in Fig. 1.

### Participant Demographics

The descriptive characteristics of the patients in the intervention (*n* = 34) and control (*n* = 33) groups are presented in Table 1. There was no significant difference between the intervention and control groups in terms of their descriptive characteristics such as age, sex, marital status, working status, education status, income status, or medications used (*p* > 0.05). The commonly used medications included beta blockers, angiotensin converting enzyme inhibitors or angiotensin receptor blockers, statins, antiplatelet agents, and nitrates.

**Table 1:** Descriptive characteristics of the patients

	Variables	Intervention (*n* = 34)	Control (*n* = 33)	Test stat.	*p*
Age (years)	X ± SD	55.74 ± 8.78	55.79 ± 6.80	t:-0.027	^d^ *0.978*
	Median (Min.-Max.)	57.5 (38–65)	56 (43–65)		
Sex, n (%)	Female	2 (5.9)	7 (21.2)	χ^2^:0.066	^a^ *0.083*
	Male	32 (94.1)	26 (78.8)		
Marital status, n (%)	Married	28 (82.4)	27 (81.8)	χ^2^:0.003	^b^ *0.954*
	Single	6 (17.6)	6 (18.2)		
Working status, n (%)	Has a regular job	17 (50)	21 (63.6)	χ^2^:6.052	^c^ *0.053*
	Has an irregular job	8 (23.5)	1 (3)		
	Not working	9 (26.5)	11 (33.3)		
Education level, n (%)	Primary school	11 (32.4)	15 (45.5)	χ^2^:2.303	^c^ *0.702*
	Middle school	9 (26.5)	5 (15.2)		
	High school	8 (23.5)	9 (27.3)		
	Undergraduate uni.	5 (14.7)	3 (9.1)		
	Graduate uni.	1 (2.9)	1 (3)		
Smoking status, n (%)	Yes	20 (58.8)	22 (66.7)		^b^ *0.680*
	No	6 (17.6)	6 (18.2)	χ^2^:0.773	
	Quit	8 (23.5)	5 (15.2)		
Alcohol consumption status, n (%)	Yes	9 (26.5)	7 (21.2)		^c^ *0.931*
	No	21 (61.8)	22 (66.7)	χ^2^:0.258	
	Quit	4 (11.8)	4 (12.1)		
Number of diseases other than hypertension, n (%)	1	19 (55.9)	16 (48.5)		^b^ *0.544*
	2 or 3	15 (44.1)	17 (51.5)	χ^2^:1.275	
Additional diseases	Diabetes	13 (38.2)	14 (42.4)		^b^ *0.727*
	Heart failure	1 (2.9)	0 (0)	χ^2^:0.122	^a^ *1.000*
	Other	1 (2.9)	2 (6.1)	χ^2^:0.985	^a^ *0.614*
Follows a diet for heart disease, n (%)	Yes	9 (26.5)	10 (30.3)		^b^ *0.728*
	No	25 (73.5)	23 (69.7)	χ^2^:0.121	
Performs exercises for one’s disease, n (%)	Yes	9 (26.5)	4 (12.1)		^b^ *0.138*
	No	25 (73.5)	29 (87.9)	χ^2^:2.205	
BMI (kg/m^2^), n (%)	Normal	6 (17.6)	8 (24.2)		^b^ *0.546*
	Overweight	16 (47.1)	11 (33.3)	χ^2^:1.351	
	Obese	12 (35.3)	14 (42.4)		
Waist circumference (cm), n (%)	Abdominal obesity (-)	14 (41.2)	14 (42.4)		^b^ *0.918*
	Abdominal obesity (+)	20 (58.8)	19 (57.6)	χ^2^:0.011	
Left ventricular ejection fraction	*X ± SD* *Median (Min-Max)*	50.44 ± 8.11 50 (40–65)	50.45 ± 8.87 50 (30–65)	t:-0.006	^d^ *0.995*
Diagnosis, n (%)	Inferior MI	15 (44.1)	13 (39.4)	χ^2^:0.829	^c^0.911
	Anterior MI	15 (44.1)	14 (42.4)		
	Posterior MI	2 (5.9)	4 (12.1)		
	Lateral MI	2 (5.9)	2 (6.1)		
PCI outcome, n (%)	Stent	30 (88.2)	31 (93.9)	χ^2^:0.668	^a^0.673
	Balloon and stent	4 (11.8)	2 (6.1)		
Vessels involved in PCI, n (%)	RCA	17 (50)	10 (30.3)	χ^2^:2.700	^b^0.100
	LAD	16 (47.1)	17 (51.5)	χ^2^:0.133	^b^0.715
	CX	4 (11.8)	8 (24.2)	χ^2^:1.773	^b^0.183
	LMCA	1 (2.9)	0 (0)	χ^2^:0.985	^a^1.000
	Diagonals	2 (5.9)	1 (3)	χ^2^:0.318	^a^1.000

^a^ Fisher’s Exact Test, ^b^ Pearson’s Chi-Squared Test, ^c^ Fisher-Freeman-Halton Test, ^d^Student’s t-Test, Max.: Maximum, Min.: Minimum, X: Mean, SD: Standard deviation, MI: Myocardial Infarction, RCA: Right Coronary Artery, LAD: Left Anterior Descending Artery, CX: Circumflex Artery, LMCA: Left Main Coronary Artery, OM: Obtuse Marginal Artery, PCI: Percutaneous Coronary Intervention

### Primary Outcomes

The results of the comparisons of the total MacNew scores of the patients are given in Table 2. The initial mean total MacNew scores of the patients were 4.76 ± 0.89 in the intervention group (range: 3.1–6.6) and 4.60 ± 0.86 in the control group (range: 3.2–5.9). There was no significant difference between the initial mean MacNew scores of the groups (*p* > 0.05). On the other hand, the total MacNew scores of the patients in the intervention group were significantly higher than those in the control group in the measurements at the end of the fourth and eighth weeks (*p* = 0.001 and *p* = 0.001; *p* < 0.01).

A significant change was observed among the initial, 4th-week, and 8th-week mean total MacNew scores of the patients in the intervention group (*p* = 0.001; *p* < 0.01). There was also a significant change among the initial, 4th-week, and 8th-week scores of those in the control group (*p* = 0.001; *p* < 0.01). While the degrees of change between the initial scores of the patients in the intervention group and their 4th-week (*p* = 0.005; *p* < 0.01) and 8th-week (*p* = 0.001; *p* < 0.01) scores were greater than the degrees of change observed in the control group (*p* = 0.005, *p* = 0.001; *p* < 0.01), there was no significant difference between the 4th-week and 8th-week scores of the patients (*p* = 0.052). The patients who performed slow breathing exercises showed increasing levels of quality of life as the follow-up period progressed, and they had higher levels of quality of life than those in the control group in all measurements.

**Table 2:** Comparisons of the total MacNew heart disease Health-related quality of life questionnaire scores of the patients

Total Score		Intervention (*n* = 34)		Control (*n* = 33)		Test stat.	*p*
**Week 0**	*X ± SD*	4.76 ± 0.89		4.60 ± 0.86		t:0.761	^d^ *0.450*
	*Median (Min-Max)*	4.8 (3.1–6.6)		4.5 (3.2–5.9)			
**Week 4**	*X ± SD*	5.90 ± 0.45		5.16 ± 0.77		Z:-4.316	^e^ 0.001**
	*Median (Min-Max)*	6 (4.9–6.7)		5.3 (3.3–6.3)			
**Week 8**	*X ± SD*	6.22 ± 0.34		5.31 ± 0.76		t:6.452	^d^ 0.001**
	*Median (Min-Max)*	6.3 (5.2–6.9)		5.3 (3.4–6.5)			
	*Test stat.*	Χ^2^:51.588		Χ^2^:20.109			
	*p*	^*f*^ 0.001**		^*f*^ 0.001**			
Diff. ∆		*X ± SD*	^*ff*^ *p*	*X ± SD*	^*ff*^ *p*		
Week 0 – Week 4		1.14 ± 0.72	0.001**	0.57 ± 0.91	0.014*	t:2.894	^d^ 0.005**
Week 0 – Week 8		1.47 ± 0.85	0.001**	0.71 ± 0.82	0.001**	t:3.691	^d^ 0.001**
Week 4 – Week 8		0.32 ± 0.31	0.007**	0.14 ± 0.42	*0.372*	t:1.980	^d^ *0.052*

^d^Student’s t-Test, ^e^Mann-Whitney U Test, ^f^Friedman Test & ^ff^Wilcoxon Signed Rank Test, Max.: Maximum, Min.: Minimum, X: Mean, SD: Standard deviation, *p < 0.05 **p < 0.01

As shown in Table 3, the GLM revealed significant effects of group allocation (*p* < 0.01) and time (*p* < 0.01) on total MacNew Questionnaire scores, and a significant group × time interaction (*p* < 0.01) indicated that changes in MacNew Questionnaire scores over time differed between the groups.

**Table 3:** General linear model results for MacNew heart disease Health-Related quality of life total scores

		F	*p*	Effect size
**MacNew Total Score**	Model	5685.960	0.001**	0.989
	Group	18.489	0.001**	0.221
	Time	112.623	0.001**	0.634
	Group * Time	13.621	0.001**	0.173

F = F-value, *p < 0.01

### Secondary Outcomes

The results of the intergroup comparisons of the heart rate, systolic blood pressure, and diastolic blood pressure values measured at home by the patients are shown in Table 4. Accordingly, the heart rate values measured by the patients in the control group at the beginning (*p* = 0.006; *p* < 0.01), at the end of the 4th week (*p* = 0.003; *p* < 0.01), and at the end of the 8th week (*p* = 0.001; *p* < 0.01) were significantly higher than those measured by the patients in the intervention group after they performed slow breathing exercises at home. While there was no significant intergroup difference between the 4th-week and 8th-week systolic blood pressure measurements of the patients (*p* = 0.1; *p* = 0.256), the initial (*p* = 0.001; *p* < 0.01), 4th-week (*p* = 0.008; *p* < 0.01), and 8th-week (*p* = 0.004; *p* < 0.01) diastolic blood pressure values of the patients in the control group were.

significantly higher than those measured by the patients in the intervention group after they performed slow breathing exercises at home. The patients who performed slow breathing exercises at home had significantly lower heart rate and diastolic blood pressure values in comparison to those who did not perform these exercises.

**Table 4:** Comparisons of the heart rate and blood pressure values of the patients

		Intervention (*n* = 34)	Control (*n* = 33)	^d^ *p*
**HR**	Week 0	70.38 ± 14.4	79.1 ± 10.15	0.006**
	Week 4	71.03 ± 11.4	79.16 ± 10.43	0.003**
	Week 8	68.59 ± 10.7	77.65 ± 10.07	0.001**
	^*g*^ *p*	*0.416*	*0.545*	
**SBP**	Week 0	121.91 ± 10.92	129.01 ± 11.89	0.013*
	Week 4	121.91 ± 13.57	127.6 ± 14.39	0.100
	Week 8	122.68 ± 14.02	126.74 ± 14.97	0.256
	^*g*^ *p*	*0.937*	*0.509*	
**DBP**	Week 0	71.26 ± 8.11	80.71 ± 11.24	0.001**
	Week 4	73.38 ± 10.34	80.49 ± 10.9	0.008**
	Week 8	73.35 ± 9.4	80.94 ± 11.39	0.004**
	^*g*^ *p*	*0.303*	*0.946*	

^d^Student’s t-Test, ^g^Repeated-Measures ANOVA & ^gg^Bonferroni Test, HR: Heart rate, Max.: Maximum, Min.: Minimum, X: Mean, SD: Standard deviation, DBP: Diastolic blood pressure, SBP: Systolic blood pressure, *p < 0.05 **p < 0.01

**Fig. 2:**
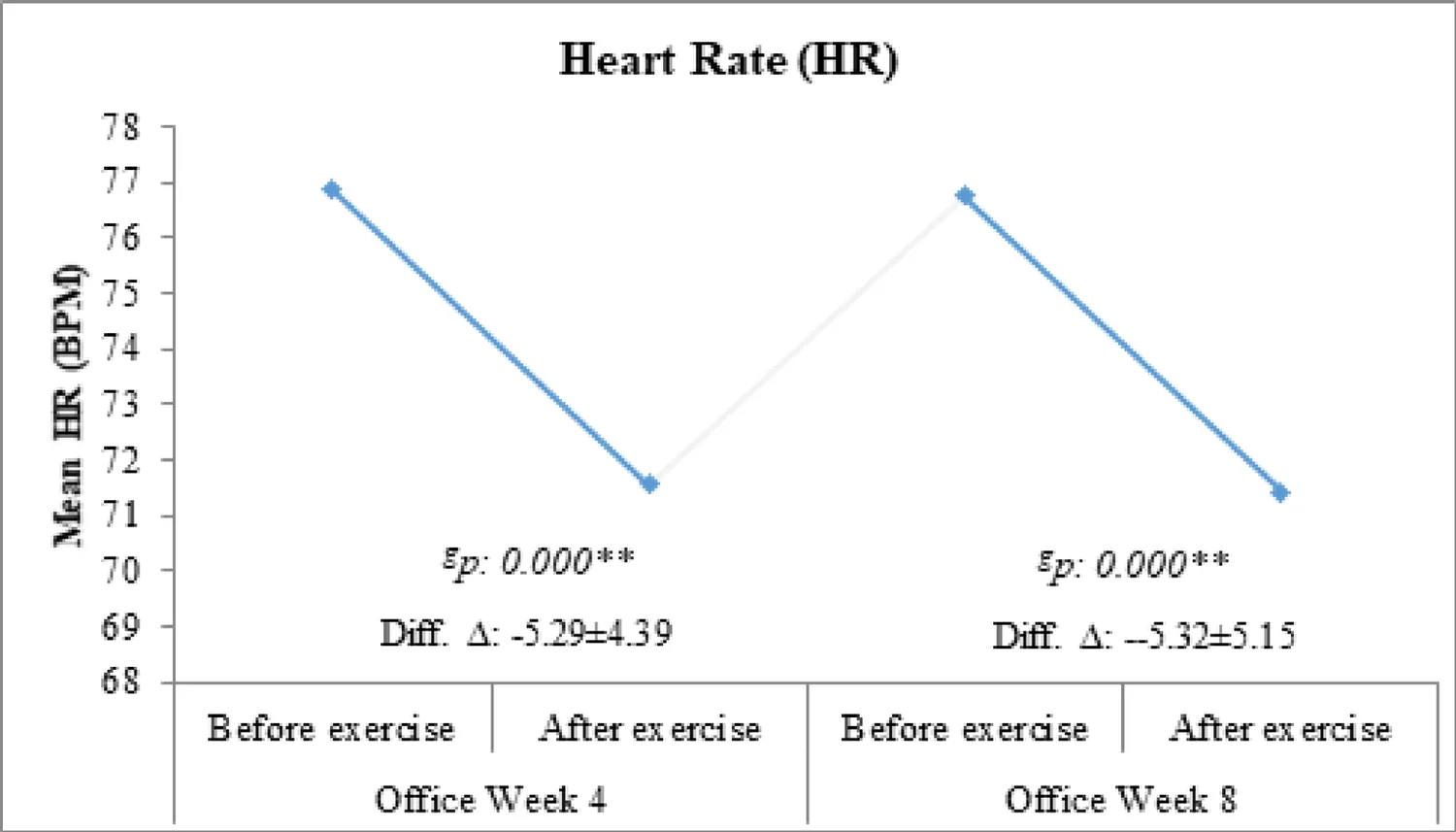
Comparisons of the pre-exercise and post-exercise heart rate values of the patients in the intervention group. Accordingly, compared to their pre-exercise measurements, after they performed the exercises, there was a mean decrease of 5.29 ± 4.39 units in the heart rate values of the patients measured in the office at 4 weeks (*p* = 0.000; *p* < 0.01), while there was a mean decrease of 5.32 ± 5.15 units in their values measured at 8 weeks (*p* = 0.000; *p* < 0.01)

**Fig. 3:**
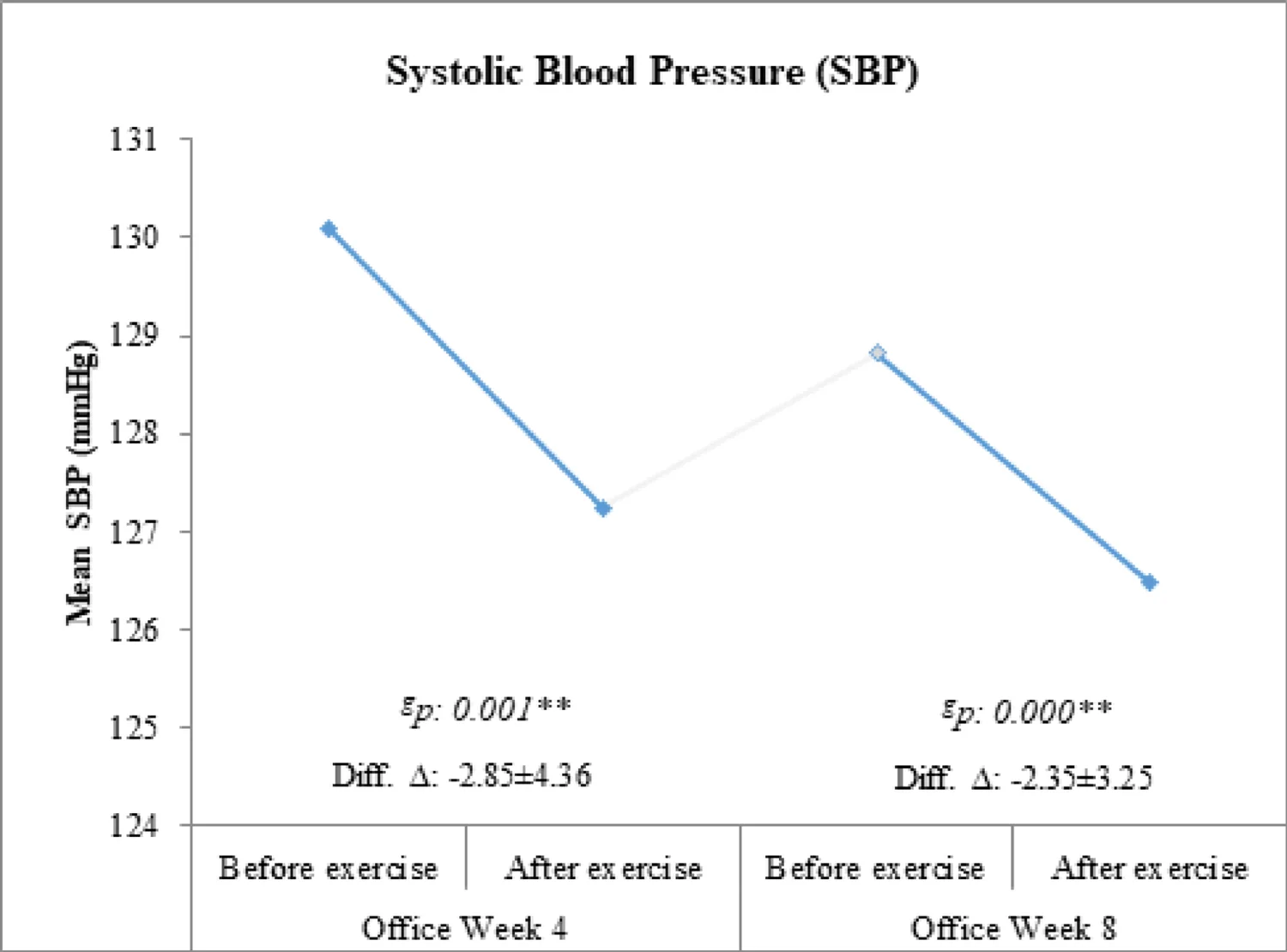
Comparisons of the pre-exercise and post-exercise systolic blood pressure values of the patients in the intervention group. In the same measurements, the mean amount of decrease in the systolic blood pressure measurements of the patients in the intervention group was 2.85 ± 4.36 mmHg at 4 weeks (*p* = 0.001; *p* < 0.01) and 2.35 ± 3.25 mmHg at 8 weeks (*p* = 0.000; *p* < 0.01)

**Fig. 4:**
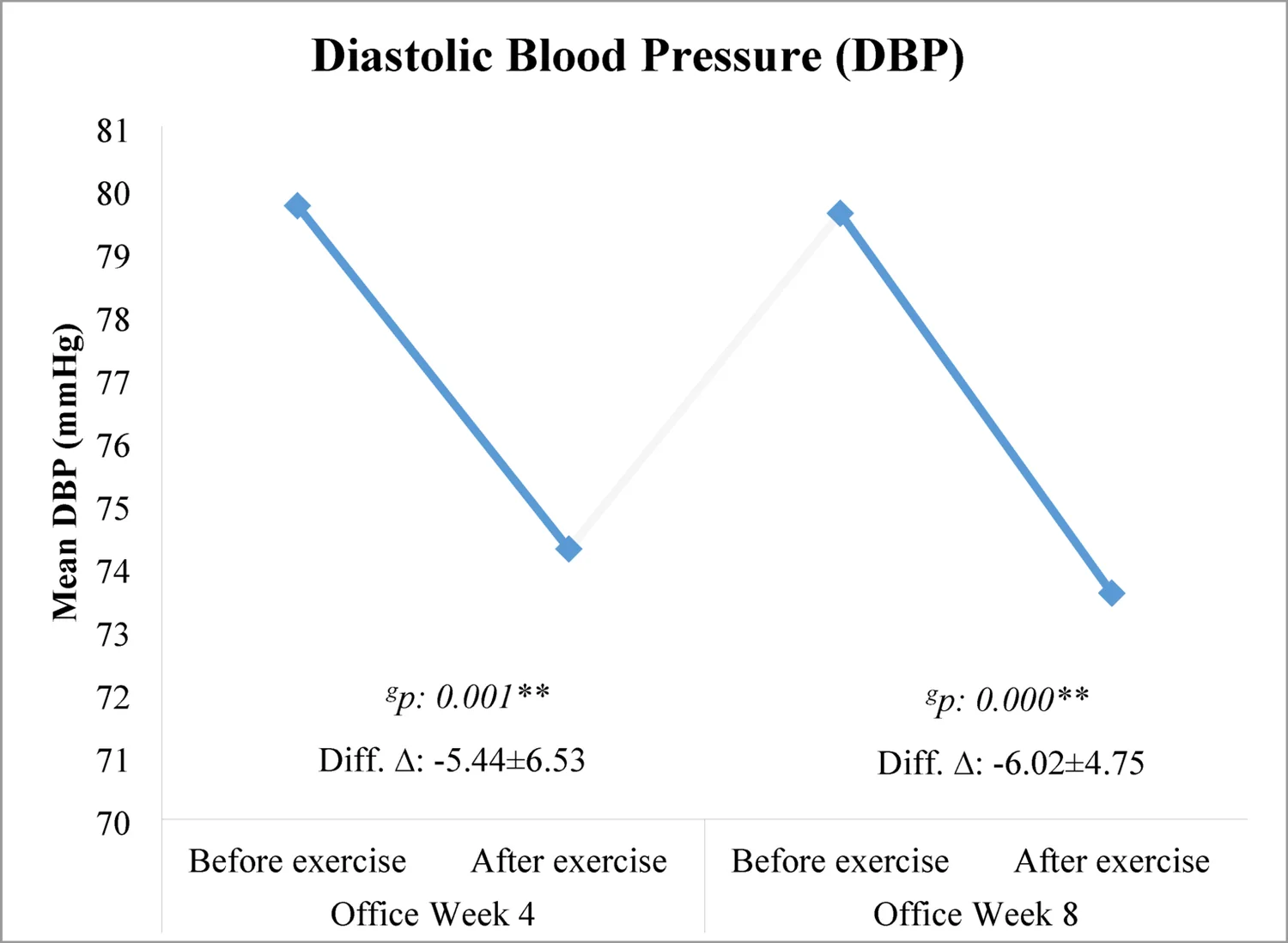
Comparisons of the pre-exercise and post-exercise diastolic blood pressure values of the patients in the intervention group. The degrees of decrease in the diastolic blood pressure values of the patients were 5.44 ± 6.53 mmHg at 4 weeks (*p* = 0.000; *p* < 0.01) and 6.02 ± 4.75 mmHg at 8 weeks (*p* = 0.000; *p* < 0.01). According to these results, slow breathing exercises led to an immediate decrease in heart rate and blood pressure values after the patients in the intervention group performed these exercises

The results of the comparisons of the heart rate and blood pressure values of the patients in the intervention group measured before and after they performed slow breathing exercises in the office are shown Figs. 2, 3 and 4.

**Table 5:** Comparisons of the heart rate and blood pressure values of patients using the generalized linear model

	Characteristic		F	*p*	Effect size
HR	Model		3928.165	0.001**	0.984
	Group		13.266	0.001**	0.169
	Time		0.017	0.897	0.000
	Group * Time		1.504	0.224	0.023
SBP	Model		8488.699	0.001**	0.992
	Group		4.283	0.042*	0.062
	Time		0.239	0.626	0.004
	Group * Time		0.973	0.328	0.015
DBP	Model		4809.582	0.001**	0.987
	Group		13.237	0.001**	0.169
	Time		1.516	0.223	0.023
	Group * Time		0.983	0.325	0.015

F: Test statistic, HR: Heart rate, DBP: Diastolic blood pressure, SBP: Systolic blood pressure, **p < 0.01 *p < 0.05

**Fig. 5:**
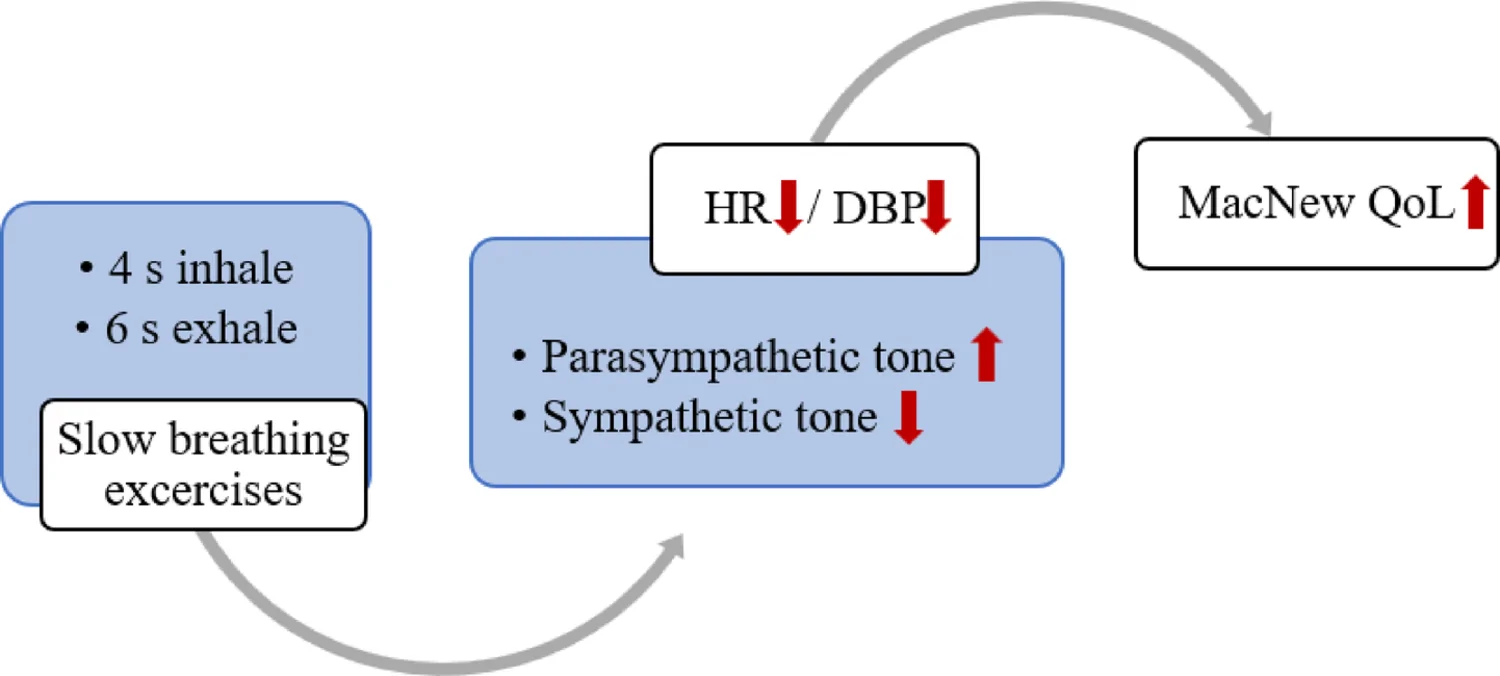
Effects of slow breathing exercises

The results of the comparisons of the heart rate and blood pressure values of the patients made using the GLM are presented in Table 5. These findings indicated that heart rate and diastolic blood pressure values were lower in the intervention group compared to the control group, whereas the difference in systolic blood pressure values was only modestly significant. The overall model for heart rate was significant (F = 3928.17, *p* < 0.01, effect size = 0.984), and the group effect was also significant (F = 13.27, *p* < 0.01), while the effects of time and the group × time interaction were not significant. For systolic blood pressure, the overall model was significant (F = 8488.70, *p* < 0.01), but the group effect was only modestly significant (F = 4.28, *p* = 0.042), with no significant time or interaction effects. For diastolic blood pressure, both the overall model (F = 4809.58, *p* < 0.01) and the group effect (F = 13.24, *p* < 0.01) were significant, whereas the effects of time and the group × time interaction were not significant. Accordingly, heart rate and diastolic blood pressure values were lower in the intervention group than in the control group. Based on these findings, the effects of slow breathing exercises are shown in Fig. 5.

### Tertiary Outcomes

Table 6 presents the drugs used by the patients in the intervention and control groups throughout the study period. During their home follow-ups, the beta blocker drug doses of 2.9% of the patients in the intervention group were raised, while the antihypertensive drug doses of 5.9% were raised. There was a significant difference between the intervention and control groups in terms of the antihypertensive drug usage statuses of the patients during their follow-ups (*p* < 0.05). The degree of increase in the antihypertensive drug doses of patients throughout their follow-up period was significantly higher in the control group than in the intervention group (χ^2^: 9.449, *p* = 0.004).

**Table 6:** Comparisons of drugs used by the patients throughout the follow-up period

		Intervention (*n* = 34)	Control (*n* = 33)	Test stat.	*p*
Beta blocker usage during follow-up, n (%)	Increased	1 (2.9)	2 (6.1)	χ^2^:0.381	^a^0.614
	No change	33 (97.1)	31 (93.9)		
Antihypertensive drug usage during follow-up, n (%)	Decreased	2 (5.9)	0 (0)	χ^2^:9.449	^c^0.004**
	Increased	2 (5.9)	11 (33.3)		
	No change	30 (88.2)	22 (66.7)		

^a^Fisher’s Exact Test, ^b^Pearson’s Chi-Squared Test, ^c^Fisher-Freeman-Halton Test, ^d^Student’s t-Test, **p < 0.01

## Discussion

In recent years, the role of quality of life in the effects of diseases and their treatments on functionality in the physical, psychological, and social fields has been emphasized. CVD cases are a special group of patients whose quality of life should be evaluated due to the physical, social, and economic effects of their conditions worldwide. Because the quality of life of an individual affects all aspects of their life, WHO recommends the usage of the term “health-related quality of life” (Spertus et al., 2004; WHOQOL, 2012). Quality of life has come to be accepted as a primary indicator in the assessments of the effectiveness of interventions made to patients, especially following MI. Previous studies have demonstrated that the health-related quality of life of patients decreases after acute MI (Brown et al., 1999; Erkan & Demirgüç, 2021; Lalonde et al., 2004; Mollon & Bhattacharjee, 2017; Szygula-Jurkiewicz et al., 2005). It has been found that poor quality of life increases repeated hospitalizations, poor long-term prognosis rates, and five-year mortality rates (Höfer et al., 2014; Kim et al., 2021; Mert et al., 2018; Pocock et al., 2021).

Breathing is not the same phenomenon as inhaling and exhaling. The manner in which a person breathes is at least as important as the quality, type, and quantity of the food they consume in their life. It is seen that breathing exercises have been used in multiple fields including the reduction of dyspnea in COPD patients, gastroesophageal reflux cases, pain management, and the alleviation of anxiety, stress, and depression (Borge et al., 2014; Chandla et al., 2013; Eherer et al., 2012; Jones et al., 2003; Park et al., 2013; Schaffer & Yucha, 2004; Sorosky et al., 2008; Valenza et al., 2014). Considering the expectation that slow breathing exercises would have an impact on the quality of life of patients undergoing primary PCI, it is believed that the information in the literature on this topic is inadequate.

The initial mean total MacNew scores of the patients were 4.76 ± 0.89 in the intervention group (range: 3.1–6.6) and 4.60 ± 0.86 in the control group (range: 3.2–5.9). This showed that the patients who were included in the study had moderate levels of heart disease-related quality of life in general (mean score: 4.7–5.10). Heart disease-related quality of life has also been reported to be moderate in other studies employing the MacNew Questionnaire (Asadi-Lari et al., 2003; Daskapan et al., 2008; Höfer et al., 2012; Nakajima et al., 2009; Pavy et al., 2015). The absence of a significant intergroup difference in the initial MacNew scores of the patients in this study (*p* > 0.05) indicated that the heart disease-related quality of life levels of the groups were initially similar. This was an important detail regarding the capacity of the results of this study to reflect the effects of the intervention over time. It was determined in this study that the patients in the intervention group had higher levels of quality of life than those in the control in all measurements made after their discharge from the hospital (*p* = 0.001; *p* < 0.01). Slow breathing exercises following primary PCI significantly improved the quality of life of the patients, as reflected by the higher total MacNew Questionnaire scores in the intervention group compared to the control group over time. The significant group-time interaction indicated that the improvement in quality of life was greater in the intervention group than in the control group. At the baseline, the intervention and control groups were homogeneous with respect to key sociodemographic characteristics, including age, sex, marital status, education level, and employment status. This homogeneity suggested that these variables would have similar effects in both groups.

This supported the hypothesis that slow breathing exercises raised the quality of life of patients. Chronic diseases and their treatments affect the quality of life of individuals as they lead to changes in their lifestyles. It was shown that breathing exercises were effective in alleviating the symptoms of asthma patients and increasing their quality of life (Santino et al., 2020; Thomas et al., 2009). In a study conducted with breast cancer patients receiving chemotherapy, deep breathing and relaxation exercises performed at home for five minutes twice a day for three weeks were found to improve the health-related quality of life of the patients (Dhillon et al., 2009).

Because there are a limited number of studies involving the performance of breathing exercises by STEMI patients undergoing primary PCI, we reviewed studies in the literature focusing on similar or shared topics for the comparisons of our results. Although we did not employ a questionnaire specifically measuring anxiety, increased anxiety could be expected to contribute to the worsening of the emotional and social aspects of quality of life after MI. According to D’silva et al. (2014), who examined the effects of deep breathing exercises performed for 10 min twice a day for two weeks by CAD patients on their anxiety and depression levels, 88.7% of the patients had anxiety, 57.5% had depression, and 7.5% had severe depression. Their results showed a positive correlation between anxiety and depression (*r* = 0.414, *p* < 0.01), while deep breathing exercises were found to significantly lower the anxiety levels of the patients (D’silva et al., 2014). Another study showed that focusing on breathing reduced stress and negative emotions, especially by activating the dorsomedial prefrontal cortex, which is a regulatory region of the brain (Doll et al., 2016). The significantly higher quality of life levels of the patients in the intervention group in our study compared to those in the control group in all measurements after their discharge from the hospital were similar to the reports in the literature. Although no scale measuring anxiety status was used in this study, these comparisons are worth noting considering the relationship between anxiety and quality of life.

Elevated heart rate and blood pressure values are closely related to certain health conditions, especially cardiovascular events. It was shown that at one- and four-year follow-ups after PCI, the mortality rates of patients whose heart rates were above 70 bpm were two times higher than the mortality rates of those whose heart rates were below 70 bpm (Antoni et al., 2012). In a meta-analysis study, it was confirmed that elevated heart rates following PCI raised mortality rates (Xu et al., 2016). Increased heart rates and high blood pressure can lead to more frequent cardiovascular events (Anchala et al., 2014; Zou et al., 2017). Many patients may find it difficult to maintain targeted heart rate and blood pressure values by using pharmacological treatments (Zou et al., 2017). For this reason, heart rate and blood pressure values are vital in CVD cases.

Breathing exercises have been utilized in many different fields such as decreasing the heart rate after PCI, controlling the heart rate and blood pressure in hypertensive patients, lowering atelectasis following surgical operations, and improving pulmonary functions (Grams et al., 2012; Jones et al., 2015; Westerdahl et al., 2005; Zou et al., 2022). Diaphragmatic breathing is a deep breathing technique shown to improve autonomic functions by reducing sympathetic activity and increasing baroreflex sensitivity (Yau & Loke, 2021).

The heart rate values measured by the patients in the control group at the beginning, at the end of the 4th week, and at the end of the 8th week were significantly higher than those measured by the patients in the intervention group after they performed slow breathing exercises at home. The systolic blood pressure values of the patients in the intervention group measured after the slow breathing exercises were also lower than those in the control group in the initial measurements. However, in the 4th-week and 8th-week measurements of the patients made at home, there was no significant difference between the systolic blood pressure values of the two groups. This discrepancy may have been caused by the adjustment made to the antihypertensive drug doses of the patients throughout the follow-up period.

The diastolic blood pressure values measured by the patients in the intervention group after they performed slow breathing exercises were lower than those measured by the patients in the control group in all measurement points. In other words, the patients who performed slow breathing exercises at home had significantly lower heart rate and diastolic blood pressure values in comparison to those who did not perform these exercises. This result supported the hypothesis that “slow breathing exercises performed after PCI reduce diastolic blood pressure.”

In a meta-analysis study, it was found that slow breathing exercises reduced the resting heart rate values of patients. In the meta-analysis, studies in which the duration of slow breathing exercise interventions varied from two weeks to six months were reviewed (Zou et al., 2017). In a study performed with HT patients, while there was a significant difference in the heart rate values of the intervention group involved in slow breathing exercises two times a week in addition to pharmacological treatment after the intervention, no significant change was seen in the control group in which patients only received pharmacological treatment (Sundaram et al., 2012). Moreover, (D’silva et al., 2014) reported differences between the heart rate values of their intervention and control groups, although these differences were not statistically significant. Another study also revealed a decrease in the heart rate values of patients in the intervention group measured at home, while the difference between the intervention and control groups was not statistically significant (Zou et al., 2022). Differences in the results of different studies in the literature may be attributed to autonomic imbalances that can be seen in HT patients.

In a previous study, the researchers that slow breathing exercises did not reduce systolic blood pressure significantly (Zou et al., 2022). However, some studies showed that breathing exercises were effective in lowering systolic blood pressure (Jones et al., 2015; Sundaram et al., 2012). Patients who performed slow breathing exercises for eight weeks by resting five seconds after each slow breath showed lower systolic blood pressure values (Jones et al., 2015). In another study involving hypertensive diabetic patients, it was determined that slow breathing exercises performed under the guidance of a device 12 min every day for 8 weeks led to significant reductions in 24-hour systolic blood pressure values (126.1 ± 3.0 vs. 123.2 ± 2.7 mmHg, *p* = 0.01) (Howorka et al., 2013). Differences in results compared to the literature may be associated with the adjustment of the antihypertensive drug doses of the patients in our study under the supervision of their physicians to prevent high blood pressure.

In this study, in the measurements of the patients made in the office when they arrived at the end of the 4th and 8th weeks for their follow-ups, the heart rate and systolic and diastolic blood pressure values of the patients in the intervention group decreased after their performance of slow breathing exercises, in comparison to their values measured before these exercises. It was shown in a previous study that performing slow and deep breathing exercises for two minutes lowered the systolic and diastolic blood pressure values of adult HT patients by 8.6 and 4.9 mmHg, respectively (Joseph et al., 2005). In another study carried out with HT patients, while there was a significant difference in the heart rate values of the intervention group involved in slow breathing exercises two times a week in addition to pharmacological treatment after the intervention, no significant change was seen in the control group in which patients only received pharmacological treatment (Sundaram et al., 2012). In an intervention group of patients performing slow breathing exercises, a significant 3.95-unit decrease was observed in heart rates measured in the office (Zou et al., 2022). The systolic blood pressure values of the intervention group of HT patients in the study performed by (Sundaram et al., 2012) who were involved in slow breathing exercises performed twice a week in addition to pharmacological treatment were lower after the intervention compared to their values before the intervention. The results of our study were similar to the results of others in the literature.

Deep breathing exercises were found effective in reducing diastolic blood pressure (D’silva et al., 2014). As a result of an intervention involving slow breathing exercises, patients with resistant HT showed significantly lower blood pressure values in measurements made both at home and in the office (Viskoper et al., 2003). In a randomized controlled study including HT patients, there was a significant difference between the intervention and control groups in terms of their diastolic blood pressure values (*p* = 0.000) (Sundaram et al., 2012). On the other hand, another study that slow breathing exercises did not reduce diastolic blood pressure significantly (Zou et al., 2022).

The antihypertensive drug doses of 5.9% of the patients in the intervention group in this study were lowered throughout their 8-week follow-up period, while the doses of 33.3% of those in the control group were raised in the same period. While the rate of patients with raised antihypertensive drug doses was higher in the control group, the rate of those with reduced or unchanged doses was lower. Throughout the follow-up period, no significant differences were found between the two groups in terms of their beta blocker drug use characteristics. Other researchers also observed a decrease in the usage doses of beta blockers in their intervention group, while the difference in this result was not significant. In the same study, a greater reduction in antihypertensive medication doses was observed in the intervention group; however, this difference was not statistically significant (Zou et al., 2022). Based on these results, one can argue that the pharmacological treatment doses of MI patients, who typically undergo intensive medical treatment, can be reduced by prescribing them with slow breathing exercises. These results indicated that slow breathing exercises constitute an effective non-pharmacological method.

### Strengths and Limitations

To avoid device-related deviations in measurement values, a blood pressure measurement device brand that has been used in previous scientific studies and is recommended by the European Society of Hypertension was used in this study. All patients were provided with the same brand and model of an automatic upper-arm blood pressure monitor (Rossmax, Model CH155f), which was procured using the project budget (Zhang et al., 2015). This standardization constituted a strong aspect of the study.

The 12-month duration of the project supervised by the Scientific and Technological Research Council of Türkiye (TÜBİTAK) was one of the limitations of the study. The project was initiated in October 2023 and ended in October 2024. However, follow-up data collection for some patients extended until November 2024 to complete the planned 8-week follow-up period. Another limitation was that the study was conducted in a single hospital in Türkiye with patients undergoing primary PCI.

The sample size was initially calculated based on Cohen’s d, requiring 70 participants. Although the inclusion of 80 participants was planned to account for potential dropouts, limitations of the predefined TÜBİTAK project timeline resulted in a smaller final sample. The results of the post hoc power analysis indicated that 58 participants (29 intervention, 29 control) were sufficient, and the study was completed with 67 participants (34 intervention, 33 control). Data from patients who withdrew from the study could not be used due to ethical considerations. It is recommended that future studies ensure data continuity to allow for Intention-to-Treat (ITT) analyses.

One of the limitations of our study was the relatively small sample size, which did not allow for subgroup or interaction analyses to assess the potential confounding effects of demographic variables such as age, sex, or smoking status. Future studies with larger sample sizes should systematically examine the potential moderating role of these demographic characteristics on the intervention effects.

## Conclusions

The main results of this study supported hypotheses H_1_: “Slow breathing exercises performed after PCI reduce heart rate,” H_3_: “Slow breathing exercises performed after PCI reduce diastolic blood pressures,” and H_4_: “Slow breathing exercises performed after PCI increase quality of life.” Slow breathing exercises may improve the quality of life of patients who have experienced MI. The findings of this pilot study suggest potential benefits for the participants included, but further research is needed to determine whether these effects are generalizable to other patient populations. Routine monitoring of quality of life after MI may be considered as part of a holistic approach to patient care.

Slow breathing exercises can be recommended as an alternative approach and lifestyle change to improve the quality of life and control the heart rate and blood pressure values of HT patients who undergo PCI following a STEMI. It is recommended that future studies with larger sample sizes explore whether sociodemographic characteristics moderate the effects of slow breathing exercises following primary percutaneous coronary intervention, to further refine patient-specific intervention strategies. More clinical studies are needed to obtain decisive evidence. The results of this study suggested that there is a need to conduct more randomized controlled studies to examine the potential benefits of this intervention in adults. It should be noted that these findings are specific to the population of this study and may not be generalized to all patients with CAD.

## Data Availability

The study protocol was registered at https://clinicaltrials.gov/ with reference number NCT06098222. The data that support the findings of this study are protected by data privacy laws and are not publicly available. However, they are available from the corresponding author upon reasonable request.
